# Association of the ApoB/ApoA1 Ratio with Angiographically Documented Coronary Artery Disease: Comparison with Conventional Lipid Markers

**DOI:** 10.3390/diagnostics16193245

**Published:** 2026-10-08

**Authors:** Tiberiu-Liviu Dragomir, Horia Silviu Branea, Minodora Andor, Ioana Mihaela Cîtu, Florina Rozina Căruntu, Monica Maria Șușan, Cristina Marinela Oprean, Raul-Tiberiu Dragomir, Răzvan Mihai Șușan, Delia Huțanu, Dan Dumitru Vulcanescu, Adriana Daniela Dragomir

**Affiliations:** 1 Medical Semiology II Discipline, Department V—Internal Medicine I, Faculty of Medicine, “Victor Babeș” University of Medicine and Pharmacy, Eftimie Murgu Square No. 2, 300041 Timișoara, Romania; dragomir.tiberiu@umft.ro (T.-L.D.); andor.minodora@umft.ro (M.A.); caruntu.florina@umft.ro (F.R.C.); 2 Multidisciplinary Heart Research Center, Faculty of Medicine, “Victor Babeș” University of Medicine and Pharmacy, Eftimie Murgu Square No. 2, 300041 Timișoara, Romania; citu.ioana@umft.ro; 3 Cardiology Clinic—ASCAR, Timișoara Municipal Emergency Clinical Hospital, Bd. Revoluției din 1989 No. 12, 300041 Timișoara, Romania; 4 Medical Semiology I Discipline, Department V—Internal Medicine I, Faculty of Medicine, “Victor Babeș” University of Medicine and Pharmacy, Eftimie Murgu Square No. 2, 300041 Timișoara, Romania; susan.monica@umft.ro; 5 ANAPATMOL Research Center, “Victor Babeș” University of Medicine and Pharmacy, Eftimie Murgu Square No. 2, 300041 Timișoara, Romania; cristina.oprean@umft.ro; 6 Department of Oncology, ONCOHELP Hospital, Ciprian Porumbescu Street No. 59, 300239 Timișoara, Romania; 7 Faculty of Medicine, “Victor Babeș” University of Medicine and Pharmacy, Eftimie Murgu Square No. 2, 300041 Timișoara, Romania; raul.dragomir@student.umft.ro; 8 Department of Family Medicine, Centre for Preventive Medicine, “Victor Babeș” University of Medicine and Pharmacy, Eftimie Murgu Square No. 2, 300041 Timișoara, Romania; razvansusan@umft.ro; 9 Department of Biology-Chemistry, Faculty of Chemistry, Biology, Geography, West University of Timișoara, Johann Heinrich Pestalozzi Street No. 16A, 300115 Timișoara, Romania; delia.hutanu@e-uvt.ro; 10 Department of Microbiology, “Victor Babeș” University of Medicine and Pharmacy, Eftimie Murgu Square No. 2, 300041 Timișoara, Romania; dan.vulcanescu@umft.ro; 11 Multidisciplinary Research Center on Antimicrobial Resistance (MULTI-REZ), Department of Microbiology, “Victor Babeș” University of Medicine and Pharmacy, Eftimie Murgu Square No. 2, 300041 Timișoara, Romania; 12 Clinical Laboratory, “Louis Țurcanu” Emergency Clinical Hospital for Children, Iosif Nemoianu Street No. 2, 300011 Timișoara, Romania; 13 Department of Allergology, Giroc Medical Center, Str. Trandafirilor No. 7, 307220 Timiș, Romania; dragomir.adriana@umft.ro

**Keywords:** ApoB/ApoA1 ratio, apolipoprotein B, apolipoprotein A1, coronary artery disease, coronary angiography, Gensini score, SYNTAX score, lipid biomarkers

## Abstract

**Background/Objectives**: The apolipoprotein B/apolipoprotein A1 (ApoB/ApoA1) ratio has been proposed as an integrated marker of atherogenic and anti-atherogenic lipoprotein balance. This study assessed its association with the presence and angiographic severity of coronary artery disease (CAD) and compared its discriminatory performance with conventional lipid markers. **Methods**: This retrospective, observational, cross-sectional study included 63 adults undergoing coronary angiography for suspected or established CAD. Pre-procedural ApoB, ApoA1, and conventional lipid parameters were measured. Significant CAD was defined as ≥50% stenosis in at least one major epicardial coronary artery. Associations with angiographic severity were assessed using Gensini and SYNTAX scores. Statistical analyses included between-group comparisons, Spearman correlations, ROC analysis with DeLong comparisons, and logistic regression. **Results**: Significant CAD was present in 46 patients (73.02%). ApoB and ApoB/ApoA1 were higher in patients with significant CAD (*p* = 0.011 and *p* = 0.013), whereas ApoA1 was not (*p* = 0.101). ApoB/ApoA1 correlated weakly with Gensini score (ρ = 0.30, *p* = 0.046), but not with SYNTAX Scores I or II. In the complete-case subset (*n* = 55), ApoB/ApoA1 had an AUC of 0.68, with no significant differences versus LDL-C, HDL-C, total cholesterol, or triglycerides. Each 0.1-unit increase in ApoB/ApoA1 was associated with higher odds of significant CAD after adjustment for age, diabetes mellitus, and smoking (adjusted OR 1.63, 95% CI 1.08–2.48; *p* = 0.021). **Conclusions**: ApoB/ApoA1 was associated with significant CAD after adjustment for selected clinical variables, but this association was attenuated after additional adjustment for LDL-C. Its discriminatory performance was modest and not significantly different from conventional lipid markers, requiring further evaluation as a potential pre-angiographic risk marker.

## 1. Introduction

Coronary artery disease (CAD) is closely associated with abnormalities in lipid metabolism, and conventional lipid parameters, particularly low-density lipoprotein cholesterol (LDL-C), remain central to cardiovascular risk assessment. However, cholesterol concentrations do not necessarily reflect the number of circulating atherogenic particles. Apolipoprotein B (ApoB) is the principal structural apolipoprotein of atherogenic lipoproteins, and its concentration reflects the number of circulating atherogenic particles, whereas apolipoprotein A-I (ApoA1) is the principal structural protein of high-density lipoprotein (HDL). The ApoB/ApoA1 ratio has therefore been proposed as an integrated measure of the balance between atherogenic and anti-atherogenic lipoprotein systems [1,2].

Large epidemiological studies support a strong association between ApoB/ApoA1 and coronary risk. In the INTERHEART study, involving more than 27,000 participants from 52 countries, ApoB/ApoA1 was the lipid-related marker most strongly associated with myocardial infarction [1]. Similarly, the AMORIS cohort demonstrated a strong relationship between elevated ApoB/ApoA1 and coronary events and suggested better predictive performance than several conventional cholesterol ratios, particularly at lower LDL-C concentrations [2]. However, this superiority has not been consistently reproduced. In the Framingham Offspring Study, ApoB/ApoA1 predicted coronary heart disease but showed discriminatory performance comparable to conventional cholesterol ratios [3]. Thus, the incremental clinical value of ApoB/ApoA1 over routinely measured lipid parameters remains uncertain.

Additional evidence supports the relationship between apolipoprotein-based measures and coronary atherosclerosis. Mendelian randomization data have shown that genetically predicted higher ApoB/ApoA1 values are associated with increased CAD risk, supporting a potentially causal relationship [4]. Moreover, in patients undergoing serial coronary computed tomography angiography, higher ApoB concentrations were independently associated with progression of coronary atherosclerosis even when LDL-C or non-HDL-C levels were relatively lower [5]. These findings suggest that particle-based lipid measures may capture aspects of atherogenic burden that are incompletely represented by cholesterol concentrations alone.

Angiographic studies have further demonstrated associations between ApoB/ApoA1 and the anatomical severity of CAD. In 6956 patients undergoing coronary angiography, Hua et al. found higher ApoB/ApoA1 values in patients with CAD and significant associations with Gensini score and multivessel disease [6]. Similar findings were reported in patients with acute coronary syndromes, in whom ApoB/ApoA1 correlated with Gensini score and showed promising discriminatory performance for severe disease [7]. Other angiographic studies have confirmed independent associations between ApoB/ApoA1, Gensini score, and the number of diseased coronary vessels [8].

Direct comparisons with conventional lipid markers also suggest potential added value. Pan et al. reported a stronger association of ApoB/ApoA1 with angiographically documented coronary disease than non-HDL-C [9]. More recently, Jiang et al., in 7277 patients undergoing coronary angiography, found ApoB/ApoA1 to be independently associated with CAD, myocardial infarction, multivessel disease, and the need for percutaneous coronary intervention, although its absolute discriminatory performance remained modest [10]. Overall, current evidence supports an association between ApoB/ApoA1 and coronary atherosclerotic burden, while its superiority over conventional lipid markers remains incompletely established.

Accordingly, the present study aimed to evaluate the relationship between the ApoB/ApoA1 ratio and both the presence and angiographic severity of CAD in patients undergoing coronary angiography. The study further assessed the association of the ApoB/ApoA1 ratio with the Gensini and SYNTAX scores and compared its discriminatory performance for significant CAD with that of conventional lipid parameters, including LDL-C, HDL-C, total cholesterol, and triglycerides.

## 2. Materials and Methods

### 2.1. Study Design

This was an observational, retrospective, cross-sectional study including adult patients who underwent coronary angiography for suspected or established coronary artery disease, in the setting of either acute coronary syndrome or chronic coronary syndrome. Clinical, biochemical, and angiographic data were retrospectively collected from the same episode of care over a period of 6 months. The study was conducted at the Ascar Cardiology Clinic, Timișoara, Romania between 1 September 2025 and 28 February 2026.

The primary biomarker of interest was the ApoB/ApoA1 ratio, calculated from pre-procedural serum ApoB and ApoA1 concentrations. Its association with the presence and angiographic severity of coronary artery disease was evaluated and its performance was compared with that of conventional lipid parameters, including total cholesterol, LDL-C, HDL-C, and triglycerides.

Blood samples were obtained prior to coronary angiography according to the local clinical workflow, generally in the fasting state as per routine practice. Individual fasting status was not systematically recorded in the retrospective dataset. Serum total cholesterol, LDL-C, HDL-C, and triglyceride concentrations were measured using routine automated clinical chemistry methods on a Dimension EXL 200 Integrated Chemistry System (Siemens Healthineers, Erlangen, Germany). ApoB and ApoA1 concentrations were measured by nephelometry using the Atellica NEPH 630 System (Siemens Healthineers, Germany). The ApoB/ApoA1 ratio was subsequently calculated from the measured ApoB and ApoA1 concentrations.

Coronary angiography was performed using a Philips Azurion 7 image-guided therapy system (Philips Medical Systems Nederland B.V., Best, The Netherlands). Coronary stenosis severity was assessed by experienced interventional cardiologists using quantitative coronary analysis (QCA) software on the Azurion system. Gensini and SYNTAX Score I were subsequently calculated by two experienced cardiologists who were blinded to clinical history and laboratory results; disagreements were resolved by consensus. SYNTAX Score II was then calculated using the original 2013 SYNTAX Score II model and the official SYNTAX Score Task Force online calculator, combining the anatomical SYNTAX Score I with the required clinical variables. Patients were additionally classified according to the presence or absence of significant coronary artery disease for comparative and ROC analyses.

### 2.2. Selection Criteria

Adult patients (≥18 years) undergoing coronary angiography for the evaluation of acute or chronic coronary syndromes were considered eligible for inclusion. Patients were required to have available pre-procedural measurements of ApoB and ApoA1 and complete angiographic data sufficient for classification of coronary artery disease status and, where applicable, calculation of the Gensini and SYNTAX scores. Conventional lipid parameters, including total cholesterol, LDL-C, HDL-C, and triglycerides, were included in comparative analyses when available.

Patients were excluded when ApoB or ApoA1 measurements were unavailable or when coronary angiography was incomplete or technically unsuitable for quantitative assessment.

For comparative analyses, patients were classified according to angiographic findings. Significant coronary artery disease was defined as a luminal stenosis of 50% or greater in at least one major epicardial coronary artery. Patients meeting this criterion were assigned to the significant CAD group. Patients in whom all major epicardial coronary arteries had stenoses of less than 50% were classified as having non-significant CAD. The 50% threshold was used as a conventional anatomical definition for the binary angiographic endpoint; left main coronary artery disease was assessed using the same threshold.

### 2.3. Ethical Considerations

The study was conducted in accordance with the Declaration of Helsinki and applicable national and institutional requirements. The study protocol received approval from the Medical Scientific Research Ethics Committee of the Timișoara Municipal Emergency Clinical Hospital (approval no. I-28215/12 August 2025). Institutional authorization for conducting the study was subsequently granted by the hospital management (no. E-3391/14 August 2025). Patient data were handled in accordance with applicable confidentiality and data-protection requirements, including Regulation (EU) 2016/679 (General Data Protection Regulation).

In accordance with applicable national regulations and institutional procedures, patients provided informed consent upon admission for the provision of medical care and for the processing and use of their medical data for healthcare-related and research purposes, as applicable. Data used in the present study were processed in anonymized or pseudonymized form and handled in accordance with the requirements of the General Data Protection Regulation (GDPR) and institutional data-protection policies.

### 2.4. Statistical Analysis

An a priori power analysis was performed using G*Power version 3.1.9.7 (Heinrich Heine University Düsseldorf, Düsseldorf, Germany). For the primary analysis assessing the association between the ApoB/ApoA1 ratio and angiographic severity of coronary artery disease, a moderate correlation coefficient of *r* = 0.35 was assumed. Using a two-sided significance level of α = 0.05 and a statistical power of 80%, the minimum required sample size was estimated at 61 participants. The final study population of 63 patients therefore met the estimated sample-size requirement for the prespecified primary correlation analysis only; this calculation was not intended to establish adequate power for the ROC comparisons or multivariable logistic regression analyses.

The distribution of continuous variables was assessed using the Shapiro–Wilk test. As most continuous variables showed non-normal distributions, they were summarized as median and interquartile range (IQR). Categorical variables were expressed as absolute frequencies and percentages.

Comparisons of continuous variables between patients with and without significant coronary artery disease were performed using the Mann–Whitney U test. Associations between continuous biochemical variables, including ApoB, ApoA1, and the ApoB/ApoA1 ratio, and angiographic severity scores were assessed using Spearman’s rank correlation coefficient (ρ). The strength of Spearman correlations was interpreted according to the absolute value of ρ as follows: <0.20, very weak; 0.20–0.39, weak; 0.40–0.59, moderate; 0.60–0.79, strong; and ≥0.80, very strong.

Receiver operating characteristic (ROC) curve analysis was performed to assess the discriminatory ability of the ApoB/ApoA1 ratio for significant coronary artery disease. The area under the ROC curve (AUC), 95% confidence interval, sensitivity, and specificity were calculated, and the optimal cut-off value was determined using the Youden index. ROC analyses were performed on the common complete-case subset The discriminatory performance of ApoB/ApoA1 was compared with LDL-C, HDL-C, total cholesterol and triglycerides using pairwise comparison of ROC curves by the DeLong method.

Univariable logistic regression analyses were performed to assess the association of individual clinical and biochemical variables with the presence of significant coronary artery disease. Multivariable logistic regression analysis was subsequently performed to determine whether the ApoB/ApoA1 ratio remained independently associated with significant coronary artery disease after adjustment for relevant covariates. Covariates were selected based on their established clinical relevance to coronary artery disease and their availability in the retrospective dataset. Given the available sample size, the number of variables included in the multivariable model was limited to reduce the risk of overfitting. Results from logistic regression analyses were reported as odds ratios (ORs) with 95% confidence intervals (CIs). For the ApoB/ApoA1 ratio, the OR was expressed per 0.1-unit increase to improve clinical interpretability. Model fit and discriminatory performance were evaluated using the likelihood-ratio χ^2^ test, Nagelkerke R^2^, the Hosmer–Lemeshow goodness-of-fit test, and the area under the receiver operating characteristic curve (AUC). Sensitivity analyses additionally evaluated adjustment for sex and, in patients with available LDL-C data, adjustment for LDL-C.

All tests were two-sided, and a *p*-value < 0.05 was considered statistically significant. Statistical analyses were performed using MedCalc Statistical Software, version 23.7 (MedCalc Software Ltd., Ostend, Belgium).

## 3. Results

### 3.1. Demographic, Clinical, Biochemical and Angiographic Characteristics

The study included 63 patients, with a median age of 65.00 years (IQR, 57.00–71.00 years). Men represented 60.32% of the cohort, while 28.57% of patients were smokers and 57.14% had diabetes mellitus. BMI data were available for 16 patients, with a median BMI of 29.12 kg/m^2^ (IQR, 22.58–30.94 kg/m^2^). The main demographic and clinical characteristics of the study population are summarized in Table 1.

The biochemical profile of the study population is summarized in Table 2. Median total cholesterol was 146.00 mg/dL (IQR, 52.50), while median HDL-C and LDL-C concentrations were 48.00 mg/dL (IQR, 15.00) and 75.40 mg/dL (IQR, 57.00), respectively. Median triglyceride concentration was 107.00 mg/dL (IQR, 70.00). Regarding apolipoproteins, median ApoB and ApoA1 concentrations were 0.77 g/L and 1.31 g/L, respectively, resulting in a median ApoB/ApoA1 ratio of 0.57. Data availability varied slightly across conventional lipid parameters, whereas ApoB and ApoA1 measurements were available for all 63 patients.

Coronary angiography identified significant coronary artery disease in 46 of 63 patients (73.02%), while 17 patients (26.98%) had no significant coronary artery disease. Among patients with significant CAD, the Gensini score was calculated in 46 patients, with a median value of 22.50 (IQR, 38.75; IQR range, 12.00–50.75). SYNTAX Score I was calculated in 45 patients, with a median value of 10.00 (IQR, 15.00; IQR range, 4.00–19.00). SYNTAX Score II was calculated for 42 patients, with a median value of 28.00 (IQR, 11.33; IQR range, 19.40–30.73). These data are observable in Table 3.

### 3.2. Association and Discriminatory Analyses

Patients with significant CAD had higher ApoB concentrations than those without significant CAD [0.80 (IQR, 0.28) vs. 0.67 g/L (IQR, 0.15), *p* = 0.011]. The ApoB/ApoA1 ratio was also significantly higher in patients with significant CAD [0.61 (IQR, 0.33) vs. 0.49 (IQR, 0.17), *p* = 0.013]. ApoA1 concentrations tended to be lower in the significant CAD group [1.26 (IQR, 0.27) vs. 1.39 g/L (IQR, 0.22)], although the difference did not reach statistical significance (*p* = 0.101). These findings are summarized in Table 4.

Among patients with significant CAD, correlations between the ApoB/ApoA1 ratio and angiographic severity were assessed in those with available data for each respective score. The ApoB/ApoA1 ratio showed a weak positive correlation with the Gensini score (*n* = 46, ρ = 0.30, *p* = 0.046), reaching statistical significance. No significant correlations were observed with SYNTAX Score I (*n* = 45, ρ = 0.18, *p* = 0.246) or SYNTAX Score II (*n* = 42, ρ = −0.07, *p* = 0.649). These findings are summarized in Table 5.

Receiver operating characteristic analysis was performed to evaluate the ability of the ApoB/ApoA1 ratio and conventional lipid parameters to discriminate between patients with and without significant CAD. To ensure direct comparability between markers, the analysis was restricted to the 55 patients with complete data for all evaluated lipid parameters, including LDL-C, of whom 39 had significant CAD and 16 had no significant CAD. Total cholesterol showed the highest individual AUC (0.75; 95% CI, 0.61–0.85; *p* < 0.001), followed by LDL-C (AUC, 0.73; 95% CI, 0.60–0.84; *p* = 0.001). The ApoB/ApoA1 ratio demonstrated an AUC of 0.68 (95% CI, 0.54–0.80; *p* = 0.015), with a sensitivity of 43.60% and specificity of 93.70% at a cut-off greater than 0.65. HDL-C and triglycerides did not individually reach statistical significance. Detailed ROC characteristics are presented in Table 6.

Pairwise comparison of ROC curves showed no statistically significant differences in discriminatory performance between the ApoB/ApoA1 ratio and any of the conventional lipid parameters. The absolute difference in AUC was 0.05 compared with LDL-C (*p* = 0.512), 0.08 compared with HDL-C (*p* = 0.256), 0.07 compared with total cholesterol (*p* = 0.438), and 0.03 compared with triglycerides (*p* = 0.735). Thus, despite numerical differences in individual AUC estimates, the discriminatory performance of the ApoB/ApoA1 ratio did not differ significantly from that of the conventional lipid markers. Pairwise AUC comparisons are presented in Table 7, while the corresponding ROC curves are shown in Figure 1.

The primary univariable and multivariable models included the full cohort (*n* = 63). In univariable logistic regression, the ApoB/ApoA1 ratio was significantly associated with the presence of significant CAD. Each 0.1-unit increase in the ApoB/ApoA1 ratio was associated with a 61% increase in the odds of significant CAD (OR, 1.61; 95% CI, 1.08–2.42; *p* = 0.021). The overall model was statistically significant (χ^2^ = 7.82, *p* = 0.005), with a Nagelkerke R^2^ of 0.17 and adequate calibration according to the Hosmer–Lemeshow test (*p* = 0.777). The model showed an AUC of 0.71 (95% CI, 0.58–0.82). Data on both univariate and multivariate analysis is displayed in Table 8.

In multivariable logistic regression analysis, the ApoB/ApoA1 ratio remained significantly associated with significant CAD after adjustment for age, diabetes mellitus, and smoking status. Each 0.1-unit increase in the ApoB/ApoA1 ratio was associated with a 63% increase in the odds of significant CAD (adjusted OR, 1.63; 95% CI, 1.08–2.48; *p* = 0.021). Age, diabetes mellitus, and smoking status were not significantly associated with significant CAD in the adjusted model. The overall model reached statistical significance (χ^2^=9.55, *p* = 0.049), with a Nagelkerke R^2^ of 0.20 and adequate calibration according to the Hosmer–Lemeshow test (*p* = 0.534). The model demonstrated an AUC of 0.73 (95% CI, 0.61–0.84). Additional adjustment for sex did not materially alter the findings, and sex was not independently associated with significant CAD (OR, 0.70; 95% CI, 0.19–2.62; *p* = 0.594).

Among patients with available LDL-C data (*n* = 55), the ApoB/ApoA1 ratio showed a moderate positive correlation with LDL-C (Spearman’s ρ = 0.596; 95% CI, 0.393–0.744; *p* < 0.001). Multicollinearity was limited, with VIF values of approximately 1.82 for ApoB/ApoA1 and 1.02 for LDL-C.

In a second sensitivity analysis restricted to patients with available LDL-C data (*n* = 55), the ApoB/ApoA1 ratio was additionally adjusted for LDL-C together with age, diabetes mellitus, and smoking status. In this model, the association of ApoB/ApoA1 with significant CAD was attenuated and was no longer statistically significant (adjusted OR per 0.1-unit increase, 1.21; 95% CI, 0.76–1.94; *p* = 0.420). The model had a Nagelkerke R^2^ of 0.28 and an AUC of 0.74 (95% CI, 0.61–0.85).

## 4. Discussion

The principal finding of this study is that the ApoB/ApoA1 ratio was associated with angiographically significant coronary artery disease (CAD), but its incremental diagnostic value was not established. The ratio and ApoB were higher in patients with significant CAD, whereas ApoA1 did not differ between groups. Each 0.1-unit increase in the ratio remained associated with significant CAD after adjustment for age, diabetes mellitus, and smoking status. However, the ratio correlated only weakly with the Gensini score, was unrelated to either SYNTAX score, and showed modest discrimination (area under the curve, 0.68) that did not differ significantly from conventional lipid markers. Its association also attenuated after LDL-C was added to the smaller complete-case model. In this cross-sectional angiography cohort, the most defensible interpretation is therefore an association with coronary atherosclerotic burden rather than proof of independent prediction or clinical superiority [4,6,10,11].

The direction of the between-group difference is consistent with several angiographic studies. Higher ApoB/ApoA1 ratios were associated with angiographic CAD or greater disease severity in cohorts reported by Hua et al. [6], Song et al. [8], Jiang et al. [10], and Jigoranu et al. [11]. An earlier Korean study also linked apolipoprotein measures with CAD in a comparatively low-risk population [12]. Direct comparisons require caution because the studies differed in enrollment setting, lipid-lowering treatment, stenosis definitions, and disease prevalence. In the present cohort, ApoB increased significantly while ApoA1 did not, suggesting that the ratio signal was driven mainly by the atherogenic-particle component. This interpretation is compatible with genetic and cohort evidence identifying the number of ApoB-containing particles as a central determinant of coronary risk [4,13,14,15].

For anatomical disease burden, the weak correlation between the ApoB/ApoA1 ratio and the Gensini score is broadly consistent with reports in which higher ratios accompanied more extensive or severe angiographic disease [6,7,8,11]. By contrast, we found no correlation with SYNTAX I or SYNTAX II, despite associations reported for related lipid or apolipoprotein indices in other acute and stable CAD cohorts [16,17].

The Gensini score weights stenosis severity and anatomical location, whereas SYNTAX I emphasizes lesion complexity and SYNTAX II additionally incorporates clinical variables. These distinct constructs may partly explain the divergent correlations. Nevertheless, the small analyzable samples for Gensini and SYNTAX scoring and the clinical heterogeneity of this cohort mean that a null correlation should not be interpreted as evidence of a true biological dissociation [16,17,18]. More broadly, recent studies have also examined circulating inflammatory biomarkers such as suPAR and hs-CRP in relation to angiographic disease severity assessed by the SYNTAX score, further illustrating that associations between blood-based biomarkers and anatomical CAD phenotypes depend on the specific biomarker, population, and angiographic endpoint [19].

A statistically significant association did not translate into strong discrimination. The ApoB/ApoA1 ratio produced an area under the curve of 0.68, and DeLong testing did not show a significant advantage over conventional lipid measures. Because only 55 participants had complete data for these comparisons, the absence of a significant difference should not be interpreted as equivalence. Earlier population studies likewise found limited or modest incremental improvement in risk discrimination after adding apolipoprotein measures to standard predictors [3,20,21]. Angiographic studies reported areas under the curve ranging from approximately 0.61 to 0.71 [22,23], while imaging cohorts have linked ApoB with coronary calcification but found variable incremental value beyond conventional lipids [24,25,26]. The observed cutoff above 0.65 combined high specificity with low sensitivity and was selected in the same dataset in which it was evaluated. It should therefore not be used to screen for or exclude CAD, or to select patients for invasive angiography, without external validation.

The attenuation of the ratio’s association after LDL-C adjustment also warrants a cautious interpretation. ApoB/ApoA1 and LDL-C were moderately correlated, and placing both variables in a small complete-case model may increase collinearity and statistical uncertainty. Loss of statistical significance does not prove the absence of an LDL-C-independent association, but these data do not demonstrate one. Serial imaging, statin-treated cohorts, and contemporary discordance analyses support ApoB as a marker of residual risk when the cholesterol content of atherogenic particles and particle number diverge [5,14,15,26,27,28,29]. That evidence primarily concerns ApoB itself rather than the ApoB/ApoA1 ratio. Together with the unchanged ApoA1 level in our study and Mendelian-randomization evidence favoring ApoB as the predominant lipid trait for coronary disease [13], this distinction argues for future head-to-head evaluation of ApoB alone versus the ratio [9,30]. In addition, intensive statin therapy can modify both conventional lipid parameters and other lipid-related biomarkers in acute coronary syndrome, underscoring the importance of detailed treatment information when interpreting ApoB-related measures [31].

Contemporary guidelines place these findings in an important clinical context. The 2019 ESC/EAS dyslipidaemia guideline, which remains the foundation of the 2025 focused update, and the 2026 ACC/AHA multisociety guideline retain LDL-C as the primary therapeutic target while supporting selective ApoB measurement when particle-cholesterol discordance or residual risk is suspected [30,32,33,34]. The 2024 ESC guideline for chronic coronary syndromes similarly emphasizes guideline-directed risk-factor and lipid management [18]. None of these documents positions the ApoB/ApoA1 ratio as a stand-alone diagnostic test for obstructive CAD or as a triage tool for coronary angiography. The ratio may therefore be a complementary research marker, particularly when conventional lipid values appear discordant, but the present results do not justify replacing established risk assessment or treatment targets.

Future studies should use prospective, consecutive, multicenter recruitment; prespecify the angiographic endpoint; document acute coronary syndrome status and lipid-lowering treatment in detail; and compare nested models containing the ApoB/ApoA1 ratio, ApoB, LDL-C, and non-HDL-C. Assessment should extend beyond association to calibration, discrimination, reclassification, decision-curve analysis, and external validation of any proposed cutoff. Until such evidence is available, our results support the biological plausibility of the ApoB/ApoA1 ratio and its association with angiographically significant CAD. However, its routine diagnostic use remains to be further studied.

### Limitations

This study has several limitations. Its observational, retrospective, cross-sectional, single-center design precludes causal inference and may limit generalizability. All participants were undergoing coronary angiography for acute or chronic coronary syndromes; the sample therefore represents a clinically selected population with an indication for invasive assessment rather than the broader population at cardiovascular risk. Because of the retrospective design, a complete screening log for all patients undergoing coronary angiography during the study period was not available; therefore, the exact number of initially screened patients and the reasons for exclusion before formation of the final analytic cohort could not be reconstructed reliably.

The modest sample size constrained the number of covariates that could be included in the regression models, and residual confounding remains possible despite adjustment for age, diabetes mellitus, and smoking. The relatively small number of patients without significant CAD also limits the stability of the multivariable logistic regression estimates and increases the possibility of model overfitting. Accordingly, the adjusted effect estimates should be considered exploratory and interpreted cautiously. Detailed information on clinical presentation, including the exact distribution of acute versus chronic coronary syndromes and prior myocardial infarction or coronary revascularization (PCI or CABG), was not consistently available in the retrospective dataset, precluding reliable stratified analyses according to clinical presentation and contributing to residual clinical heterogeneity. Similarly, lipid-lowering treatment was incompletely documented, including statin use and intensity and the use of ezetimib or PCSK9 inhibitors. Consequently, the potential influence of background lipid-lowering therapy on ApoB, ApoA1, and conventional lipid concentrations could not be adequately assessed and may have contributed to the relatively low LDL-C values observed in this cohort. In patients with acute coronary syndrome, the interval between symptom onset and blood sampling was not systematically available. BMI was also not systematically documented in the retrospective records and was available for only 16 of 63 patients, precluding its meaningful inclusion in multivariable or subgroup analyses.

Comparative ROC analyses were restricted to 55 complete cases, and the Gensini and SYNTAX analyses used smaller subsets. SYNTAX Scores I and II were unavailable in a small number of patients because complete information required for score calculation could not be consistently retrieved retrospectively. The a priori sample-size calculation was based on the primary correlation analysis and indicated a target of 61 participants; however, the angiographic severity analyses were restricted to 42–46 patients with available scores, and the comparative ROC analysis to 55 complete cases. These secondary analyses were therefore likely less well powered than the original sample-size calculation implied, particularly for SYNTAX Scores I and II. Accordingly, null correlations and nonsignificant pairwise AUC differences should not be interpreted as evidence of no association or equivalence. Formal interobserver agreement statistics were not calculated; therefore, although disagreements were resolved by consensus, residual interobserver variability cannot be excluded.

The ROC-derived cutoff should be considered exploratory and requires external validation. Because biomarkers and angiographic findings were assessed during the same episode of care, the results describe association and discrimination rather than prospective prediction or causality.

Despite these limitations, the study provides a direct comparison between ApoB/ApoA1 and conventional lipid markers in angiographically documented disease. Larger prospective multicenter studies should evaluate atherosclerosis progression and cardiovascular outcomes, test incremental value beyond ApoB, LDL-C, non-HDL-C, and established clinical predictors, and prespecify any candidate cutoff. More complete recording of clinical presentation, prior myocardial infarction and coronary revascularization, as well as the type and intensity of lipid-lowering therapy, would also permit stronger multivariable and subgroup analyses.

## 5. Conclusions

In this retrospective study of patients undergoing coronary angiography, the ApoB/ApoA1 ratio was significantly higher in patients with significant coronary artery disease and remained significantly associated with significant CAD after adjustment for age, diabetes mellitus, and smoking status. Each 0.1-unit increase in the ApoB/ApoA1 ratio was associated with a 63% increase in the odds of significant CAD. However, this association was attenuated and was no longer statistically significant after additional adjustment for LDL-C.

The ApoB/ApoA1 ratio also showed a weak positive correlation with Gensini score, whereas no significant associations were observed with SYNTAX Scores I or II. Its discriminatory ability for significant CAD was modest, and although its AUC did not differ significantly from those of LDL-C, HDL-C, total cholesterol, or triglycerides, superiority over conventional lipid markers was not demonstrated.

These findings support an association between the ApoB/ApoA1 ratio and the presence and angiographic burden of coronary artery disease in patients referred for coronary angiography. Larger prospective and multicenter studies are required to determine whether ApoB/ApoA1 offers incremental value beyond conventional lipid markers and to clarify its potential role in pre-angiographic risk stratification.

## Figures and Tables

**Figure 1:**
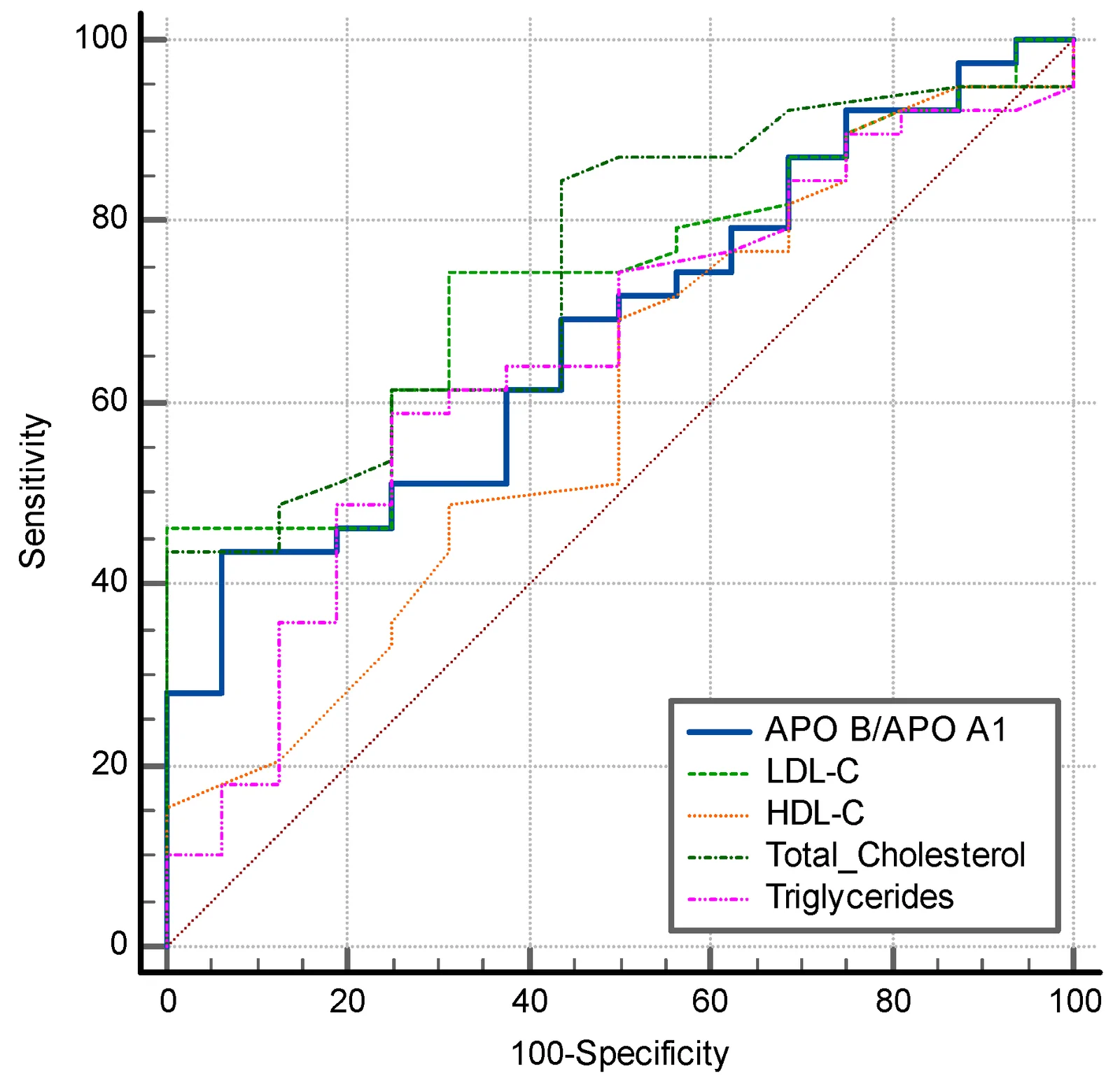
Receiver operating characteristic curves for ApoB/ApoA1 and conventional lipid markers in discriminating significant coronary artery disease among patients with complete lipid data (*n* = 55).

**Table 1:** Demographic data.

Variable	Overall Population	Significant CAD (*n* = 46)	No Significant CAD (*n* = 17)	*p*-Value **
Age, years, median (IQR)	65.00 (57.00–71.00)	64.50 (56.00–70.00)	68.00 (60.00–73.00)	0.242
BMI, kg/m^2^, median (IQR) *	29.12 (22.58–30.94)	29.12 (23.22–30.85)	26.72 (24.12–29.32)	0.812
Male sex, *n* (%)	38 (60.23%)	28 (60.87%)	10 (58.82%)	1
Smoking, *n* (%)	18 (28.57%)	13 (28.26%)	5 (29.41%)	1
Diabetes mellitus, *n* (%)	36 (57.14%)	27 (58.70%)	9 (52.94%)	0.777

*: BMI data were available for 16 of 63 patients in the entire lot, for 14/46 patients with significant CAD, for 2/17 patients without significant CAD. **: continuous variables were compared using the Mann–Whitney U test; categorical variables using Fisher’s exact test.

**Table 2:** Laboratory values for the studied lot.

Variable	Available n	Median	IQR	IQR Range
Total cholesterol, mg/dL	62/63	146.00	52.50	126.50–179.00
HDL-C, mg/dL	62/63	48.00	15.00	41.00–56.00
LDL-C, mg/dL	55/63	75.40	57.00	56.80–113.80
Triglycerides, mg/dL	62/63	107.00	70.00	80.25–150.25
ApoB, g/L	63/63	0.77	0.27	0.66–0.93
ApoA1, g/L	63/63	1.31	0.30	1.19–1.49
ApoB/ApoA1 ratio	63/63	0.57	0.29	0.46–0.74

**Table 3:** Angiographic scores.

Variable	Calculated n (Significant CAD)	Median	IQR	IQR Range
Gensini score	46/46	22.50	38.75	12.00–50.75
SYNTAX Score I	45/46	10.00	15.00	4.00–19.00
SYNTAX Score II	42/46	28.00	11.33	19.40–30.73

**Table 4:** Comparison of apolipoprotein parameters according to the presence of significant coronary artery disease.

Variable	Significant CAD (*n* = 46), Median (IQR)	IQR Range	No Significant CAD (*n* = 17), Median (IQR)	IQR Range	*p*-Value
ApoB, g/L	0.80 (0.28)	0.70–0.98	0.67 (0.15)	0.62–0.77	0.011 *
ApoA1, g/L	1.26 (0.27)	1.18–1.45	1.39 (0.22)	1.32–1.54	0.101
ApoB/ApoA1 ratio	0.61 (0.33)	0.49–0.82	0.49 (0.17)	0.41–0.58	0.013 *

*: *p* < 0.05, statistically significant.

**Table 5:** Correlation between the ApoB/ApoA1 ratio and angiographic severity scores in patients with significant CAD.

Correlation	n	Spearman ρ	*p*-Value
ApoB/ApoA1 vs. Gensini score	46	0.30	0.046 *
ApoB/ApoA1 vs. SYNTAX Score I	45	0.18	0.246
ApoB/ApoA1 vs. SYNTAX Score II	42	−0.07	0.649

*: *p* < 0.05, statistically significant.

**Table 6:** ROC analysis of ApoB/ApoA1 and conventional lipid markers for the identification of significant CAD (*n* = 55).

Marker	AUC	95% CI	Cut-Off	Sensitivity, %	Specificity, %	*p*-Value
ApoB/ApoA1 ratio	0.68	0.54–0.80	>0.65	43.60	93.70	0.015 *
LDL-C, mg/dL	0.73	0.60–0.84	>91.40	46.20	100.00	0.001 *
HDL-C, mg/dL	0.60	0.46–0.73	≤54.00	69.20	50.00	0.242
Total cholesterol, mg/dL	0.75	0.61–0.85	>173.00	43.60	100.00	<0.001 *
Triglycerides, mg/dL	0.65	0.51–0.78	>109.00	59.00	75.00	0.060

*: *p* < 0.05, statistically significant.

**Table 7:** Pairwise comparison of ROC AUCs between the ApoB/ApoA1 ratio and conventional lipid markers.

Comparison	Absolute ΔAUC vs. ApoB/ApoA1	*p*-Value
ApoB/ApoA1 vs. LDL-C	0.05	0.512
ApoB/ApoA1 vs. HDL-C	0.08	0.256
ApoB/ApoA1 vs. total cholesterol	0.07	0.438
ApoB/ApoA1 vs. triglycerides	0.03	0.735

**Table 8:** Univariable and multivariable logistic regression analyses for significant CAD.

Model	Predictor	OR	95% CI	*p*-Value
Univariable	ApoB/ApoA1, per 0.1-unit increase	1.61	1.08–2.42	0.021 *
Multivariable	ApoB/ApoA1, per 0.1-unit increase	1.63	1.08–2.48	0.021 *
	Diabetes mellitus	1.08	0.32–3.68	0.906
	Smoking	0.58	0.14–2.38	0.449
	Age, per year	0.96	0.90–1.03	0.240

*: *p* < 0.05, statistically significant.

## Data Availability

Data is available upon request from the corresponding author.
